# A Brief Review of Genetic Approaches to the Study of Food Preferences: Current Knowledge and Future Directions

**DOI:** 10.3390/nu11081735

**Published:** 2019-07-26

**Authors:** Antonietta Robino, Maria Pina Concas, Eulalia Catamo, Paolo Gasparini

**Affiliations:** 1 Institute for Maternal and Child Health, IRCCS “Burlo Garofolo”, Via dell’Istria 65/1, 34137 Trieste, Italy; 2 Department of Medical Sciences, University of Trieste, Strada di Fiume, 447, 34149 Trieste, Italy

**Keywords:** genetics, food preferences, heritability, candidate gene, GWAS, adiposity, polygenic risk score

## Abstract

Genetic variation plays a crucial role in individual differences in food preferences which ultimately influence food selection and health. Our current understanding of this pathway has been informed through twin studies (to assess the heritability of food preferences), candidate gene studies, and genome-wide association studies (GWAS). However, most of this literature is mainly focused on genes previously identified as having taste or smell functions. New data suggests that genes not associated with taste or smell perception may be involved in food preferences and contribute to health outcomes. This review highlights these emerging findings and suggests a polygenic risk assessment approach to explore new relationships between food preferences and health risks.

## 1. Introduction

Food preferences are shaped by a high number of environmental, cultural, and nutritional factors, including genetic ones. The first evidence for genetic influences on food preferences came from family and twin studies [1,2,3,4,5,6,7,8,9,10,11,12]. However, over the last few decades, rapid advances in molecular genetics have revolutionized the understanding of individual differences in many aspects of human behavior. These advances give researchers the tools to conduct genetic association studies on a large scale to better understand the role of specific gene loci in sensory perceptions, food liking/disliking, preference, and intake, as well as on food-related habits [13,14,15,16,17,18].

To date, the vast majority of studies on food liking and preference have focused on identifying specific genes and traits associated with sensory perceptions (mainly taste and smell perception). The effects of taste and smell genes on food habits [19,20,21,22,23,24,25,26,27,28,29,30,31,32,33,34,35,36] and health status [30,31,37,38,39,40,41,42,43,44,45,46,47,48] have also been extensively investigated. However, gaps in understanding still exist, and emerging evidence suggests that novel genes (not necessarily related to taste or smell perception) may play a critical role in these relationships [13,14,15,16].

Thus, a potential new area in nutrition research is the investigation of the genetic bases of food preferences, broadly defined to include both taste/smell-related and non-related genes. 

Obtaining a comprehensive picture of genetic effects on food preferences and habits and their consequences for food-related diseases, such as being overweight or obesity, is of considerable public health importance and interest to the food industry.

This review focused on current knowledge, linking genetic variability to food preferences. Specifically, we reviewed studies on food preferences (defined as the selection of one food rather than another) and food liking (meaning the degree of liking or disliking towards a food). 

## 2. Genetic Dissection of Food Preferences

The genetic background of a trait can be investigated through several methods. Firstly, heritability analysis allows one to estimate the proportion of variation of a phenotype, which is due to genetic differences between individuals. However, heritability studies do not provide any information on specific genes and polymorphisms related to a given trait. Specific information can be identified through genetic association analysis such as candidate gene and genome-wide approaches. A candidate gene study investigates variations within specific genes of interest selected on the basis of existing knowledge or hypotheses. In contrast, a Genome-Wide Association Study (GWAS) is conducted without suppositions or previous knowledge and the whole genome is scanned so that new genetic variants may be discovered [49,50,51]. 

Here, we report different approaches through which the genetics of food preferences can be dissected. Firstly, we review studies that provide evidence for a genetic basis of food preferences (heritability studies) and then studies that identified underlying genes (candidate genes and genome-wide association). Finally, we describe the possible relationships between genes linked to food preferences and health status, and we present an example of the predictive power of polymorphisms associated with vegetable liking on adiposity measures.

### 2.1. Heritability Studies

Heritability is the proportion of the phenotypic variation in a population explained by genetic effects; it is a measure of the inheritance of a trait. Usually, heritability estimation requires data where familial relationships are known (twins or family studies) and does not provide information about which genes are responsible for the trait. Heritability has been widely estimated in twin studies, where monozygotic twins (identical twins with almost no differences in their DNA) are compared to dizygotic twins (fraternal twins who share, on average, half of their DNA). This comparison allows one to evaluate the proportion of variation of a trait ascribable to genetic factors, while the remaining variance is assumed to derive from environmental factors. Heritability estimation ranges from 0 to 1: a high value indicates that genetics plays a major role, while low values indicate that most of the variation is due to environmental factors. High heritability does not necessarily imply that a single gene is the cause of trait variation. It is possible that multiple genes, each of them having a small effect, contribute to this variation [52].

Evidence on the heritability of food preferences has been reported in both adult and children twin studies. For example, studies of 3–5 year-old children provide evidence for high or moderate heritability for liking of vegetables (from 0.37 and 0.54), fruits (from 0.51 to 0.53), and proteins (from 0.48 to 0.78) [4,5]. Moderate heritability for specific food preferences such as vegetables (0.54), fruits (0.49), meat or fish (0.49), and dairy (0.44) has also been observed in adolescents (18–19 years of age) [6]. Similar findings have been reported in adults. In a cohort of ~600 adult female twins in the UK, Keskitalo and colleagues reported that 0.49, 0.54, and 0.53 of the variation in liking for a sweet solution, liking and use-frequency of different sweet foods (sweet desserts, sweets, sweet pastry, ice cream, hard candy, and chocolate), respectively, was explained by genetic factors [8,9]. Similarly, a study in young adult Finnish twins showed that genetic effects account for 0.18–0.58 of the variation in the pleasantness of oral pungency, spicy foods, and pungent sensations [10]. In the same cohort, genetic influences on sour foods were studied, and 0.14 and 0.31 of the variation in pleasantness and intensity of orange juice spiked with citric acid was reported [11]. Moreover, these same authors also found that genetic effects accounted for 0.34–0.50 of the variation in pleasantness and use-frequency of sour foods categorized into three groups as follows: sour fruits and berries (red currant, red currant juice, cranberry, lingonberry, lemon, and rhubarb), sour dairy products (natural cultured milk, natural yogurt, and sour milk), and less-sour berries and fruits (strawberry, orange, blueberry, peach, and banana) [11]. 

Differences in heritability results across studies can be explained by the small sample size of most studies and by the minimal number of foods analyzed (i.e., different from study to study and mainly focused on the taste perception of foods). Moreover, differences in the data collection and analysis (i.e., age differences of participants, use of different questionnaires and measurements, analysis of single foods or a set of clustered foods) could also be responsible for this variability.

More recently, a large study of more than 2000 UK twins analyzed the heritability of different liking patterns using data from an online food liking–disliking questionnaire including 87 different foods and beverages. This study revealed four food-liking patterns by principal component analysis (PCA): fruit and vegetables; sweet and high carbohydrates; meat; distinctive tastes (including chili pepper, garlic, or other foods with strong taste). Moderate heritabilities were obtained for all of them (fruit and vegetables: 0.36; sweet and high carbohydrates: 0.52; meat: 0.44; distinctive tastes: 0.58), corroborating past works on genetic influences of food liking–disliking **[12]**. However, similar heritability estimates reached by studies with both large and small sample size suggest that environmental factors also play a crucial role. 

Overall, these studies are useful in providing a quantitative estimate of the heritability of food preferences and in supporting the idea that genetic determinants play a role. However, as already mentioned, they do not give information concerning specific genes accounting for food preferences.

### 2.2. Candidate Gene Studies

A candidate gene study requires an “a priori” hypothesis based on a potential role of a given gene on a given trait of interest [53]. Regarding food preferences, this approach has been used to examine the possible role of polymorphisms in genes already known to be involved in taste or smell perceptions. These two senses allow us to recognize and to discriminate foods and are among the most important determinants of food liking/disliking [54,55,56]. For these reasons, DNA polymorphisms in taste and smell genes have played an important role in individual variability on food choices.

#### 2.2.1. Taste Receptor Genes

It is well known that genetic factors influence taste perception. Genes encoding taste receptors have been identified and genetic variability of sweet, umami, and bitter perceptions have been intensely investigated, although knowledge gaps exist for sour and salty perception [19,20,21,22,23,24,25,26,27,28,29,30,31,32,33,34,35,36,37]. As stated above, comprehensive reviews have already been published on the relationship between variations in taste receptors and food preferences [38,39,40,41,42,43,44], thus in the present review, we only present a few examples.

A very well-known example is that of the *TAS2R38* bitter receptor, a major contributor to individual differences in bitter taste perception of PROP (6-n-propylthiouracil) or PTC (phenylthiocarbamide). About 30%–40% of the European population is taste-blind to these compounds or perceive them as weakly bitter (so-called non tasters), while the remaining 70%–60% can perceive them as moderately or intensely bitter (so-called tasters). Three SNPs (Single Nucleotide Polymorphisms) in the *TAS2R38* gene (rs1726866, rs10246939, rs713598) result in three amino acid substitutions defining two main haplotypes, namely AVI and PAV, that confer differences in the ability to taste PTC/PROP. Indeed, individuals homozygous for the AVI haplotype are mainly non tasters, while homozygous for the PAV haplotype and heterozygous individuals are likely to be tasters [19,20,57,58]. 

Although controversial results have emerged in the literature, the variation in the ability to perceive PROP has been widely related to preferences for different foods such as brassica vegetables, other bitter foods, sweets, added fat, spicy foods, and alcoholic beverages [37,38,59,60,61,62]. For example, Mennella and collaborators showed that in children, but not in adults, *TAS2R38* variations partially explained individual preferences for sucrose or beverages and cereals with a high sugar content [63]. A study in Malaysian adults showed mixed results. Specifically, they reported that aversions to individual foods such as green tea, mayonnaise, and whipped cream were associated with *TAS2R38* genotypes, while no associations were observed for vegetables and sweet/fatty foods [64]. More recently, a study by Shen et al. showed that AVI/AVI subjects liked brassica vegetables more than PAV/AVI and PAV/PAV individuals [65]. In another recent work, Perna and collaborators reported that one specific polymorphism in the *TAS2R38* gene was associated with preferences for beer, butter, and cured meat [66]. However, a link between *TAS2R38* genetic variants and food liking has not been observed in other studies and several reasons could be responsible for the inconsistent findings such as food assessment methods, sample size, cultural habits, or other environmental factors that may influence the association.

Evidence for a relationship between other bitter taste receptor genes and liking of common foods and beverages have also been reported. For example, variation in the *TAS2R19* bitter-taste gene showed associations with grapefruit juice bitterness and liking [37], while another bitter-taste gene, *TAS2R43,* has been related to coffee liking [67]. Data also suggested a possible influence of genetic variation in the *TAS1R3* sweet receptor gene on sweet preferences in children [68], as well as a link between variations in the *CD36* gene (responsible for fat taste perception) and fat preferences [31].

The studies reviewed above have limited implications for general food preferences because they only analyze one or few genes (or SNPs) and they examine liking for just one or few foods. To address this shortcoming, our group examined the relationship between a broad spectrum of food preferences and DNA variants in several taste and olfaction genes in a large cohort of >400 individuals. Statistically significant associations were identified for genes involved in chemosensory functions (i.e., *TRPV1* and *TAS1R2*) or in signal transduction (i.e., *PLCβ2* and *ITPR3*). One of the most interesting associations was found between the *TAS1R2* gene (coding for a sweet taste receptor) and liking of alcoholic beverages, according to data reporting a link between ethanol preference and liking for sweet taste. Specifically, the lower frequent allele for two different SNPs (rs3935570 and rs4920566) in the *TAS1R2* gene were positively associated with the liking of vodka and white wine. Another noteworthy association was detected for tea and the *PLCβ2* gene, a marker for type II taste bud cells, which is involved in the caffeine response and is also expressed in the sensory cells of the olfactory epithelium. In this case, the rarest allele of rs2290550 SNP was negatively correlated with tea liking [15].

#### 2.2.2. Olfactory Receptor Genes

Humans vary in their capacity to perceive several odors, and their variation in olfactory receptor (OR) genes may be responsible for these differences [69,70]. Despite more than 400 genes/receptors being involved in smell perception, little is known about the link between these genes and specific odorants as well as their possible influence on food preferences. One of the most recognized examples is the role of the olfactory receptor gene *OR7D4*, which is partially responsible for individual differences in the ability to smell androsterone [69]. Androsterone is undetectable for some people, others define it as foul smelling or urine and sweat smelling, while others describe it as sweet or floral smelling. Two SNPs in the *OR7D4* gene are responsible for two amino acid substitutions that impair the ability to perceive androstenone [70]. Androstenone is present in the meat of male pigs. A recent study confirmed that *OR7D4* variants were associated with the sensory perception of pork meat containing androstenone as well as lower liking for the flavor and odor of pork meat by androstenone-sensitive individuals [71].

Another example is the *OR2J3* gene, which is associated with individual differences in detecting *Cis*-3-hexen-1-ol (C3HEX), an odorant with a green/grassy smell and is present in several fruits and vegetables. Polymorphisms in this gene are responsible for amino acid substitutions impairing the ability to smell C3HEX. Subjects can be classified as C3HEX-sensitive or C3HEX-insensitive [72,73]. Moreover, foods spiked with C3HEX were less acceptable than the unspiked foods; however, the reductions in acceptability were more marked in C3HEX-sensitive individuals if compared to C3HEX-insensitive individuals [74]. 

Finally, studies examined variation in the *OR5A1* gene, related to β-ionone odor sensitivity. β-ionone aroma is a fruity/floral aroma that is present in several foods and beverages [75,76,77,78]. A series of studies by Jaeger and co-workers showed that a DNA variation (rs6591536 SNP) in the *OR5A1* gene is the causal variant for β-ionone odor sensitivity, explaining 96.3% of the phenotypic variation. They also reported that β-ionone sensitive individuals can easily differentiate between foods (such as milk chocolate or apple juice) with and without added β-ionone, and they can also recognize β-ionone in foods when compared to less-sensitive individuals. Moreover, sensitive individuals prefer foods without β-ionone rather than with β-ionone [79].

### 2.3. GWA Studies

Over the past decade, the GWAS approach has become one of the most common tools for the identification of genes associated with complex traits and diseases. In these studies, a large number of participants are genotyped for a large number of genetic markers (usually SNPs) covering the whole genome and their relationships with the trait of interest are examined, allowing for the identification of novel gene variants and genomic loci [80].

To date, very few GWAS have been conducted on food preferences, which are summarized in Table 1. Although a genome-wide scan typically analyzes thousands or even millions of SNPs, Table 1 reports only GWAS significant SNPs with *p*-value < 5 × 10^−8^. This *p*-value is equivalent to the Bonferroni-corrected threshold (α = 0.05) for 1 million independent variants (approximately the number of independent SNPs analyzed in a GWAS).

The first GWAS was carried out on cilantro (or coriander) liking in a large cohort of unrelated European subjects belonging to the 23andMe cohort [81], who responded to an online questionnaire asking whether they taste cilantro as soapy and whether they like it. An association among the rs72921001 SNP, soapy taste, and disliking of cilantro was found. This SNP falls within a cluster of eight olfactory receptor genes on chromosome 11. Among them, the authors suggested that a good candidate for cilantro preferences could be the *OR6A2* gene coding for a receptor that can be activated by several aldehydes responsible for the characteristic odor of cilantro [18]. 

More recently, we conducted the first GWAS on red and white wine preference assessed by survey-reported food liking in 3885 adults coming from different geographic areas (Italy, the Netherlands, and Central Asia). In this work, we detected a significant association between white wine liking and rs9276975 SNP in the *HLA-DOA* gene, encoding for a non-canonical MHC (major histocompatibility complex) II molecule. Although the mechanism of how MHC could be linked to wine preferences is unknown, the possible involvement of the olfactory system was hypothesized [16]. Moreover, another GWAS on the liking of 20 different foods was carried out on a large cohort of 4611 individuals, which identified 15 novel significant variants associated with 12 different foods. Some of these variants are located within genes that might represent good candidates for food choices. Interestingly, none of them belong to taste or olfactory receptor gene families, but are likely to be involved in reward response to food (i.e., *BPNT1*, *IRX4*, *CNTN5*, and *CSMD1* genes). For example, an association was detected between the liking of bacon and rs140738262 SNP in the *CNTN5* gene. This polymorphism also showed marginal association with the liking of other fatty foods such as lamb, pork chops, and goat cheese. This gene is expressed in the brain and has previously been associated with anorexia nervosa, suggesting a possible link with preferences for palatable food and the responsivity of the brain reward system to these foods. For vegetables, an association between chicory liking and rs138369603 SNP in the *CSMD1* gene has emerged. We hypothesized a possible role of this gene in the regulation of the food reward response since its variants were linked to differential activation of the cuneus, an area possibly involved in central reward processing [17]. 

Overall, these results represent a step in understanding the biological bases of food liking and suggest that the GWAS approach may be useful in identifying novel candidate genes for food preferences. Nowadays, thanks to the reduction of SNP genotyping costs as well as to the existence of large population biobanks, GWA studies could contribute to identifying many more loci, which will enhance insight into the genetic architecture of food preferences. Thus, further studies should be conducted to confirm previous findings, to extend the range of examined foods, and to also analyze other food groups.

## 3. From genetic Variations in Food Preference Genes to Health

There is a well-developed body of research examining the relationships between taste receptor genes and their downstream effects on food preferences and intake, which may in turn affect nutritional and health status [31,43,44,45,46,47,48]. These studies are reviewed elsewhere [82], however a few salient examples are discussed here. For instance, SNPs in the *TAS1R2* and *TAS1R3* genes, which codify for sweet taste receptors and are related to a higher preference and intake of sweet foods, have also been associated with increased dental caries [46,83,84]. Another example is the relationship between variations in the *TAS2R38* bitter taste gene and eating behavior as well as anthropometric and adiposity measures. Increased disinhibition has been described in women carrying the PROP-insensitive allele for the rs1726866 SNP [85]; while another finding reported higher BMI and waist circumference among PROP non-taster women with low dietary restraint [86]. In another study, differences in body fat percentage were associated with the three *TAS2R38* genetic variants, while no significant relationships with BMI and eating behavior were found [87]. Other studies did not support a relationship between *TAS2R38* variants and adiposity measures [64,86,87,88]. These inconsistent results could be ascribed to the presence of several confounding factors (i.e., sex, age, ethnicity, etc.) that may modulate the relationship among taste receptors and health status parameters. 

Differences in bitter taste perception have also been associated with bitter taste receptor mRNA levels in taste cells [89,90], suggesting that gene expression is another factor to consider when the relationship with health measures is studied. Moreover, recent findings showed that the gene expression profile of fungiform taste papillae differs between lean and obese subjects [91]. Together, these findings highlight the need to conduct future studies to clarify their association. 

Recent evidence also raises the possibility that taste and smell receptors residing in different bodily tissues may have multiple functions in health and disease. For example, taste receptors are also expressed in extra-oral tissues, such as the gastrointestinal tract, where they seem to be involved in digestive functions or homeostasis and energy metabolism [92,93,94,95,96,97,98,99,100,101,102,103].

It is also well known that the sense of smell is impaired in neurodegenerative diseases [104,105] and associations between olfactory genes (expressed in olfactory and non-olfactory tissues) and diet-related diseases such as obesity have also been demonstrated [104,106,107]. Notably, the *OR7D4* gene, recently related to preference for pork meat containing androstenone (described in [71]), was previously associated with adiposity, cognitive dietary restraint, and susceptibility to hunger in another study [108]. 

Despite these positive findings, very large GWAS on BMI or other health-related parameters have not found associations with SNPs in chemosensory genes [109,110,111], suggesting that their effects are likely to be very small and limited in predictive power.

### Combining Several Genetic Variants: The Polygenic Risk Score

The evidence presented above suggests that a new paradigm may be needed to accelerate progress in understanding the relationships between food preferences and nutrition and health. Our findings [17] using the GWAS approach identified novel genes associated with food preferences with no known effects on chemosensory function. Thus, looking beyond the involvement of traditional chemosensory genes in food preferences may be important for gaining new insights.

Although GWA studies have led to progress in identifying common variations associated with many complex traits, the modest effect sizes have prevented risk prediction based on single genetic variants. More recently, polygenic risk score analyses that combine the effects of several genetic variants have shown some predictive ability for a wide range of complex traits [112]. In polygenic score (PGS) analysis, a set of SNPs identified in a GWAS is used to construct a polygenic score that is used for association testing or risk prediction.

To the best of our knowledge, polygenic risk score analyses for food preferences have not yet been conducted. Although the link between vegetable intake and adiposity measures was widely investigated [113,114,115], few studies focusing on the relationship between hedonic measures and adiposity have been conducted. These studies have found no or weak association [116,117,118], suggesting that this complex relationship could be modulated by several factors, including genetic ones. Therefore, here, we report the data obtained from a PGS analysis to evaluate the predictive power of SNPs associated with food liking on adiposity measures (BMI and fat mass). Data was collected from 1140 individuals belonging to two Italian cohorts (Friuli Venezia Giulia and Val Borbera). Further details on data collection, sample characteristics, and polygenic score analysis are reported in supplementary materials (File S1).

We constructed a PGS for vegetables (PGS-vegetables) based on 6 SNPs significantly associated with preferences for different vegetables in our previous work: rs28849980, rs10050951, rs8034691 for artichokes, rs2530184, rs9832668 for broccoli, and rs138369603 for chicory (see Table 2 in [17]). 

For each individual, PGS-vegetables represents vegetable preference predicted by the combination of the above mentioned 6 SNPs. In the first step, the allele count for each SNP was weighted by its per-allele association with food preferences. Specifically, for each identified SNP, an individual’s genotypic score (0, 1, or 2 for genotyped SNPs, or any value between 0–2 for imputed SNPs) was multiplied by the effect size. SNPs were weighted such that a higher weight was associated with a higher preference for the associated vegetable. The final score (PGS-vegetables) was calculated for each individual by summing the values obtained in the first step across all six SNPs. Linear regression analysis was conducted to test the associations between adiposity measures (BMI and fat mass as dependent variables) and PGS-vegetables as the predictor variable, in models adjusted for sex, age, education level (as number of years of completed schooling), and physical activity (never/light/moderate/intense). Information on sample collection, genotyping, imputation, and phenotypes were reported in our previous works [16,17]. Table 2 shows that PGS-vegetables was a significant negative predictor of BMI and fat mass (*p*-value < 0.05), in addition to sex, age, education, and physical activity. Specifically, higher PGS-vegetables (corresponding to higher preferences for vegetable foods) was predictive of lower BMI and fat mass.

Although the PGS-vegetables variable accounted for only 0.28% of the variation in BMI and 0.33% of the variation in fat mass, the low number of SNPs included in the study could explain this finding. 

These results on PGS represent a starting point in studying the polygenic effects of food preferences on health status. As the number of GWAS of food preferences increase, further studies considering more SNPs and other food categories should be conducted. Adopting the PGS approach would allow the development of more powerful genetic profiles to better predict the risk of disease. 

## 4. Conclusions

In conclusion, the data reviewed here highlight the role of genetic variations in food preferences and their important contributions to nutrition and health.

There is a need to identify and investigate other genes involved in food preferences, besides those already implicated in olfactory and taste perception. These novel genes can be discovered through GWAS or other genomic approaches.

The use of polygenic risk analysis to assess associations between food preferences and disease outcomes could lead to important new insights in nutrition research.

## Figures and Tables

**Table 1:** GWA studies of food liking.

Reference	Subjects (*n*)	Population	Food Liking Assessment	Associated Trait	SNP	Locus
Eriksson et al., 2012 [18]	26,455	Unrelated (European)	Responses to an online survey asking the following questions: - Does fresh cilantro taste like soap to you?” (Yes/No/I’m not sure) - Do you like the taste of fresh (not dried) cilantro?” (Yes/No/I’m not sure)	Cilantro	rs72921001	*OR6A2*
Pirastu et al., 2015 [16]	3885	Isolated population (European and Central Asia)	Survey-reported food liking (5-point scale or 9-point scale)	White wine	rs9276975	*HLA-DOA*
Pirastu et al., 2016 [17]	4611	Isolated population (European and Central Asia)	Survey-reported food liking (5-point scale or 9-point scale) (5-point scale or 9-point scale)	Artichokes	rs28849980	CCRN4L
				Artichokes	rs28849980	ADAMTS19-CHSY3
				Artichokes	rs8034691	LOC100128714
				Broccoli	rs2530184	NA
				Broccoli	rs9832668	RYBP
				Broccoli	rs138369603	CSMD1
				Bacon	rs140738262	CNTN5
				Oil or Butter on Bread	rs6661761	BPNT1
				Blue Cheese	rs12994253	KCMF1-TCF7L1
				Ice Cream	rs2035613	IRX4
				Liver	rs34088951	RNU6-66
				Coffee	rs145671205	FIBIN

Associated trait refers to the associated food liking; SNP column shows the name polymorphism; Locus column refers to the gene closest to the most significant SNP; GWA refers to Genome-Wide Association.

**Table 2:** Results of polygenic risk score analysis.

Predictor Variables	BMI, Kg/m^2^	Fat Mass, Kg
Sex, male	**2.85 (<0.0001)**	−0.67 (0.2)
Age, years	**0.04 (<0.0001)**	**0.09 (<0.0001)**
Education level, years	**−0.14 (<0.001)**	**−0.26 (0.002)**
Physical activity	**−1.19 (<0.0001)**	**−2.56 (<0.0001)**
Vegetables PGS	**−0.98 (0.028)**	**−2.08 (0.023)**

Beta and *p*-value in brackets are shown. In bold: significant results (*p*-value < 0.05). BMI = Body Mass Index; PGS = Polygenic Score.

## Supplementary Materials

The following are available online at https://www.mdpi.com/2072-6643/11/8/1735/s1, File S1: Data collection and polygenic score analysis.
