# Supplementary material for: A Brief Review of Genetic Approaches to the Study of Food Preferences: Current Knowledge and Future Directions
Source: Nutrients. 2019 Jul 26;11(8):1735. doi: 10.3390/nu11081735 (PMC6722914; doi:10.3390/nu11081735)
Supplement: Supplementary file 1 [file nutrients-11-01735-s001.pdf]

Data collection and polygenic score analysis

A total of 1140 individuals belonging to the Italian Network of Genetic Isolates (INGI) were used in this study for calculation of polygenic risk score (PGS): 706 coming from six villages located in the Friuli Venezia Giulia (FVG) Region in Northern-Eastern Italy and 434 from the Val Borbera (VB) Valley in Northern-Western Italy. A full description of these samples has been previously reported [1,2]. All participants gave written informed consent, and the ethical committees of IRCCS Burlo Garofolo and San Raffaele approved the study.

Personal information, such as physical activity level (never/light/moderate/intense) and educational level (elementary (5 years), lower secondary (3 years), upper secondary (5 years), university (5 years)) were assessed in each participant by standard questionnaires.

BMI and fat mass were measured by the Bioelectrical Impedance Analysis technique using the Body fat Composition Analyzer (Tanita BC-420MA; Tanita, Tokyo, Japan).

Table 1. shows the characteristics of samples used in the present PGS analysis.

Table A1. Sample characteristics.

	Cohort	
	Friuli Venezia Giulia	Val Borbera
Number of samples	706	434
Females, %	60.74	39.26
Age, years mean (standard deviation)	52 (16.4)	58.7 (15.2)
Education level, years mean (standard deviation)	10.6 (3.6)	10.5 (4.1)
Physical activity, %		
Never	13.2	19.4
Light	29.3	28.6
Moderate	45.3	46.5
Intense	12.2	5.5
BMI, Kg mean (standard deviation)	25.5 (4.9)	25.1 (4.1)
Fat Mass, Kg mean (standard deviation)	20.9 (9.5)	19.6 (8.5)

Genotyping was carried out with Illumina 370k high-density SNP array. Genotype imputation was conducted after standard quality control using a custom reference panel integrating Whole Genome Sequence data available for INGI samples with resources from the 1000 Genomes project using the method implemented by the IMPUTE2 software [3].

Statistical analyses for PGS calculation were carried out with R 3.3.0. (www.r-project.org).

For this analysis 6 SNPs previously associated at genome wide significance p-value (p-value<$5x10^{-8}$) with liking of different vegetables were selected [4]. Specifically, artichokes liking resulted associated with rs28849980 (β=–0.052), rs10050951 (β=0.031) and rs8034691(β=0.040), broccoli liking with rs2530184 (β=–0.048) and rs9832668 (β=–0.127) and chicory liking with rs138369603 (β=0.084).

For each SNP, the individual's genotypic score (0, 1, or 2 for genotyped SNPs or any value between 0-2 for imputed SNPs) was extracted and for each individual this value was multiplied by the effect size (β) of the SNP. All SNPs were coded according to higher preference for the associated vegetable. Then, for each individual a PGS-vegetables was the summed values obtained for each SNP, as follow:

$$PGS\text{-}vegetables = \beta_1 {*} X_1 + \beta_2 {*} X_2 + \ldots + \beta_6 {*} X_6$$

in which β_i is the effect of SNP_i on the vegetable and X_i is the genotypic score of the SNP_i.

In Figure S1 the distribution of PGS-vegetables by sex (A) and by population (B) are shown.

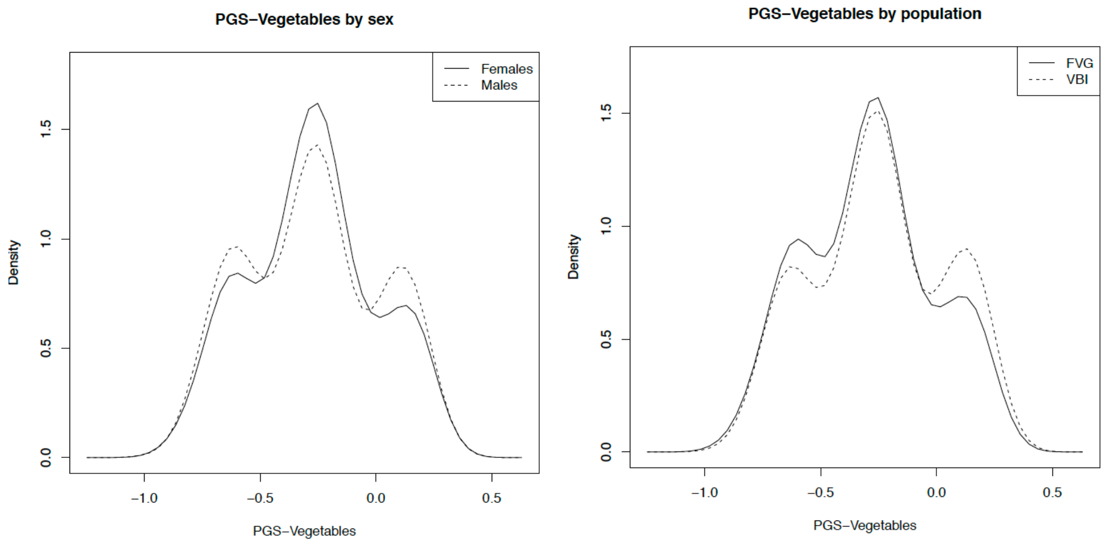

A) B)

Figure S1. PGS-vegetables by sex (A) and population (B).

Next, this PGS-vegetables was used to evaluate its effect on adiposity. Two linear regression models were performed where PGS-vegetables was used as predictor and BMI or fat mass as dependent variables. Analyses were adjusted for sex, age, education level and physical activity.

Because similar effects were observed in FVG and VB samples, the results reported in Table 2 refer to the analysis performed on the whole sample.

Supplementary references

1. Esko, T, Mezzavilla, M., Nelis, M., Borel, C., Debniak, T., Jakkula, E., … D'Adamo, P. Genetic characterization of northeastern Italian population isolates in the context of broader European genetic diversity. European Journal of Human Genetics 2013 21(6), 659–665.
2. Xue, Y, Mezzavilla, M., Haber, M., McCarthy, S., Chen, Y., Narasimhan, V., Zeggini, E. Enrichment of low-frequency functional variants revealed by whole-genome sequencing of multiple isolated European populations. Nature Communications 2017 8, 15927.
3. Howie, B., Marchini, J., & Stephens, M. Genotype imputation with thousands of genomes. G3 (Bethesda, Md.) 2011 1(6), 457-70.
4. Pirastu N, Kooyman M, Traglia M, Robino A, Willems SM, Pistis G, Amin N, Sala C, Karssen LC, Van Duijn C, Toniolo D, Gasparini P. A Genome-Wide Association Study in isolated populations reveals new genes associated to common food likings. Rev Endocr Metab Disord. 2016 17(2), 209-19.
